# An evaluation of the Eppendorf EPOS 5060 biochemistry autoanalyser

**DOI:** 10.1155/S1463924687000178

**Published:** 1987

**Authors:** F. Antoja, M. J. Alsina, M. T. Casamajó, C. Ribera, R. Galimany

**Affiliations:** Servicio de Análisis Clínicos Centro de Asistencia Primaria ‘Dr Robert’ Badalona (Barcelona) Spain


**Journal of Automatic Chemistry**/*Journal of Clinical Laboratory Automation*, **Vol. 9, No. 2 (April–June 1987), pp. 77–81**

### An evaluation of the Eppendorf EPOS 5060 biochemistry autoanalyser

**F. Antoja, M. J. Alsina, M. T. Casamajó, C. Ribera and R. Galimany**

*Servicio de Análisis Clínicos, Centro de Asistencia Primaria 'Dr Robert', Badalona (Barcelona), Spain*

### Introduction

The EPOS 5060 is a selective batch analyser for routine and special work in clinical chemistry, with a theoretical throughput of 300 tests/h.

The evaluation reported here was done according to the protocol of the Sociedad Española de Química Clínica (SEQC) [1].

Different components of the analyser were evaluated for representative analytes: creatinine, total protein, glucose, AST and alkaline phosphatase; comparing results with those from the Ultrolab-Aurora analyser [2].

### Materials and methods

*The instrument*

The EPOS 5060 is manufactured by Eppendorf Gmbh (Hamburg, FR Germany) and is distributed in Spain by Merck-Igoda, S.A.

It consist of a single module (70 × 110 × 70 cm), and it weighs approximately 140 kg. All measuring procedures and calculations are completed in the one unit.

A data processor (6410) is available for rapid request input, work list preparation (also for additional analysers) and the preparation of patient reports and quality-control data. The present evaluation did not include the data processor.

The reaction rotor consist of 20 quartz glass cuvettes with a 10 ± 0·01 mm lightpath. Temperature is controlled through a Peltier system and is adjustable between 20 and 40 °C (±1 °C). An appropriate washing solution is used for cleaning and drying by a filtered air system.

Measurement is done with a monochromatic spectral line photometer, equipped with a mercury lamp and double interference filters. Wavelengths used are 334, 405, 492, 546 and 578 nm, with amplifications of 365, 436 and 623 nm.

The samples are placed into coded micro-test tubes with numerical identification.

Up to 30 different methods may be memorized, with one or two reagents available for each. Reagent 1 is contained in a vessel with 80 ml maximum volume, whose cover is formed in such a way that it can take, at any one time, up to six standards of different concentrations, three control sera and a cleaner. These may also be set up on the sampler chain. The reagent 2 container is located above the rotor and is positioned automatically according to the preselected times. Its maximum volume is 20 ml.

Since the instrument operates in batch mode, the reagent containers must be changed between different methods. At the beginning of a method the dispensing system operates always for filling and washing the tube system.

Dispensing volumes are freely selectable within the preselected ranges and are set automatically. The dispensers use a Hamilton pipetting system, with plastic tubes for reagent 1 and metallic tubes for reagent 2 and sampler.

The sample dispenser drifts down the sample tube lid before suction to avoid vacuum effects. Its range is between 2·5 and 50 µl, adjustable in steps of 0·25 µl.

The reagent dispensing system has a range between 200 and 500 µl, in steps of 1·0 µl for reagent 1, and between 5 and 50 µl, adjustable in steps of 0·25 µl for reagent 2.

Mixing takes place in the reaction cuvette by a compressed air system.

The last calibration is stored – so it is unnecessary to calibrate at each process batch.

*Reagents*

The following reagents were used:

For the mechanical evaluation:

p-nitrophenol (PNP) (Sigma 104–8); NaOH (Merck 6498).

From a solution of 0·36 mmol/l of PNP in NaOH (20 mmol/l) different concentrations were obtained by dilution.

b-nicotinamide adenine dinucleotide, reduced form (NADH) disodium salt (Sigma N8129); tris-(hydroxymethyl)aminomethane (Merck 8382).

All the other solutions were prepared from 1 mmol/l solution of NADH in tris 80 mmol/l.

For tests on samples of sera:

Merckotest creatinine (Merck 3385); Merckotest total protein (Merck 3327); Merckotest glucose GLUC-DH (Merck 14335); Merckotest AST (TRIS IFCC) (Merck 14346); Merckotest alkaline phosphatase (Merck 3344).

For correlation with the Ultrolab-Aurora:

Testomar creatinine combipack (Behring OVPC 10/11); Testomar total protein combipack (Behring OSOA 10/11); Cromatest glucose (Hexokinase) (Knickerbocker 250 B106); Testomar ASAT (GOT) IFCC (Behring OUCZ 10/11); Testomar alkaline phosphatase (DEA) (Behring ORSP 20/21).

For the calibrator:

Aurora calibrator (Behring OUDO 20/21).

For control sera:

Ortho serum control normal assayed (Ortho Diagnostics); Ortho serum control abnormal assayed (Ortho Diagnostics).

### Parameters evaluated

*Photometric inaccuracy*

Photometric accuracy was studied at 340 nm with a solution of disodium NADH (333 μmol/l) in Tris buffer (80 mmol/l), and at 405 nm with PNP solution (143·8 mmol/l) in NaOH (20 mmol/l) [3 and 4]. Dilutions were prepared manually. Up to 30 consecutive measurements were made for each absorbance, using the same cuvette.

Inaccuracy was calculated from the experimental values and the theoretical values obtained from the coefficient of molar absortivity of NADH and PNP.

*Photometric imprecision*

From solutions prepared as previously, 30 successive measurements were obtained in the same cuvette, and from these were calculated the mean, standard deviation and coefficient of variation at both 340 and 405 nm [3].

*Photometric linearity*

Using serial dilutions prepared as before, three successive determinations were made for each absorbance, always in the same cuvette [4]. Theoretical absorbances were calculated from the coefficient of molar absortivities of NADH and PNP.

*Photometric drift*

Photometric stability was studied over the first 30 min and at a free 12 h at 405 nm with PNP in NaOH solution of theoretical absorbance 1·00. Three successive determinations were made in the same cuvette.

*Sample pipette delivery system imprecision*

The reagent 1 delivery system was set to dispense a constant amount of NaOH (20 mmol/l), and the sampler (sample pipette) to dispense volumes ranging from 2 to 50 μl of PNP solution. In each case, final absorbance was arranged to be around 0·5. Standard deviation and coefficient of variation were calculated from 30 determinations [5].

*Reagent pipettes delivery system imprecision*

The sampler pipette was blocked and NaOH (20 mmol/l) dispensed by the reagent 1 pipette, and PNP solution by the reagent 2 pipette.

Five experiments were carried out with volumes between 100 and 300 μl for the reagent 1 pipette, and 1 to 60 μl for the reagent 2 pipette. Standard deviation and the coefficient of variation were calculated from 30 successive determinations [5].

*Temperature control*

Warm-up time was studied making readings every 15 s, until three consecutive readings with a deviation of ±1 °C were obtained. 30 readings were then made at 20 s intervals for 10 min. The mean and variance were calculated.

*Imprecision*

Within the same run, 30 samples of control sera were tested at three levels, in order to study the within-run imprecision. To evaluate between-run imprecision, a further 30 samples were distributed in different runs.

*Carry-over*

Following a permutation order, according to the Comisión de Instrumentación de la SEQC [1], three control samples with different concentrations were distributed along the sample chain.

*Relative inaccuracy*

Using 100 samples from patients, results from the EPOS 5060 were compared with those from the ULTROLAB-AURORA, applying regression analysis.

*Sample dilution system inaccuracy*

Using the glucose assay, previously measured sera were reassayed using the following dilutions: 1/1, 1/2 and 1/5.

### Results and discussion

*Photometric inaccuracy*

The photometric inaccuracy for NADH solution at 340 nm, expressed as percentage accuracy was −5·24% for 1·463 absorbance, and +3·92% for 0·053 absorbance. For PNP solution at 405 nm it was −8·10% for 0·919 absorbance and +3·20% for 0·516 absorbance (see table 1).

*Photometric imprecision*

The coefficients of variation ranged from 0·10 to 1·83% at 340 nm; at 405 nm they ranged from 0·16 to 2·10% (see table 2).

*Photometric linearity*

The obtained linearity is right for NADH at 340 nm and for PNP at 405 nm. The results are shown in figures 1 and 2. There is some dispersion at low absorbances for both reagents.

*Table 1. Photometric inaccuracy; N = 30.*

| NADH (240 nm) Theoretical absorbance | Observed absorbance | Inaccuracy (%) | PNP (405 nm) Theoretical absorbance | Observed absorbance | Inaccuracy (%) |
|---|---|---|---|---|---|
| 2·057 | 2·019 | −1·84 | 2·000 | 1·968 | −1·60 |
| 1·544 | 1·463 | −5·24 | 1·500 | 1·497 | −0·02 |
| 1·029 | 1·024 | −0·48 | 1·000 | 0·919 | −8·10 |
| 0·514 | 0·489 | −4·86 | 0·500 | 0·516 | +3·20 |
| 0·205 | 0·209 | +1·95 | | | |
| 0·051 | 0·053 | +3·92 | | | |

*Table 2. Photometric imprecision; N = 30.*

| NADH (340 nm) Mean absorbance | CV (%) | PNP (405 nm) Mean absorbance | CV (%) |
|---|---|---|---|
| 2·019 | 0·10 | 1·981 | 0·16 |
| 1·463 | 0·41 | 1·488 | 0·21 |
| 1·024 | 0·21 | 0·994 | 0·25 |
| 0·489 | 0·29 | 0·505 | 0·18 |
| 1·194 | 0·36 | 0·204 | 1·55 |
| 0·054 | 1·83 | 0·055 | 2·10 |

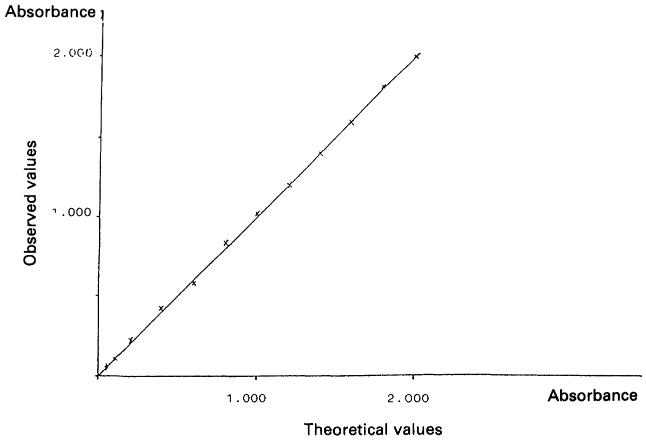

*Figure 1. Photometric linearity; NADH (340 nm).*

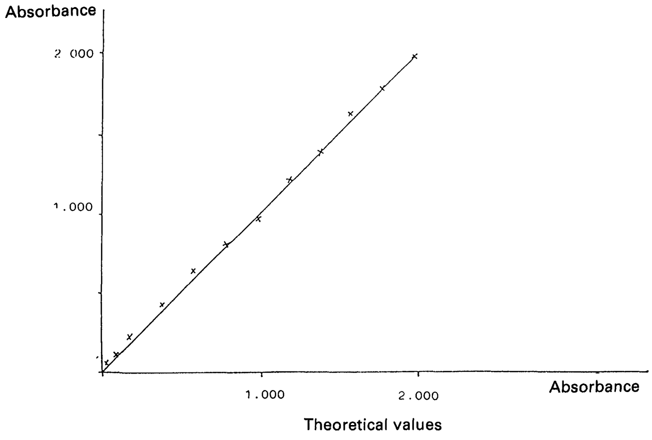

*Figure 2. Photometric linearity; PNP (405 nm).*

*Photometric drift*

For the photometric stability test, PNP was read at 405 nm for 30 min, using a solution with a mean absorbance of 1·094. The coefficient of variation obtained was 0·24%. With the same solution read at 12 h, the mean absorbance was 1·039 and the coefficient of variation was 0·60%.

*Pipette delivery system imprecision*

For the reagent pipette, an imprecision of 0·78% was found for the largest volume for both the first and second reagents. For the smaller volumes, imprecision was 0·20. Reagent volume therefore affects imprecision (see table 3). Sample pipette imprecision is also directly related to the volume dispensed (see table 3).

*Table 3. Imprecision of the pipette delivery system; N = 30.*

| Reagent pipette 1 (μl) | Reagent pipette 2 (μl) | CV (%) |
|---|---|---|
| 300 | 1 | 0·78 |
| 100 | 60 | 0·30 |
| 300 | 5 | 0·87 |
| 235 | 15 | 0·47 |
| 190 | 60 | 0·20 |
| **Sample pipette (μl)** | **Reagent pipette 1 (μl)** | **CV (%)** |
| 2 | 250 | 2·14 |
| 5 | 250 | 1·98 |
| 10 | 250 | 1·55 |
| 25 | 250 | 1·24 |
| 50 | 250 | 1·18 |

The photometric imprecision can be considered to be negligible when compared with the delivery system.

*Temperature control*

Testing each 15 s, warm-up times were found to be as follows: to attain 25 °C = 240 s; to attain 30 °C = 150 s; to attain 37 °C = 330 s.

Findings for attained temperature were as follows: theoretical temperature: 25 °C/experimental temperature: 24·9 °C; 30 °C/29·9 °C; 37 °C/36·7 °C.

The variances found were smaller than those of the thermometer used.

*Imprecision*

Within-run and between-run imprecision is shown in table 4.

*Carry-over*

Results are shown in table 5. For practical purposes, no carry-over is apparent.

*Relative inaccuracy*

The results are shown in table 6 and figures 3 and 4.

*Sample diluting system imprecision*

The following coefficients of variation were obtained:

| Sample dilution | CV (%) |
|---|---|
| 1/1 | 2·0 |
| 1/2 | 3·4 |
| 1/5 | 5·8 |

*Table 4. Overall imprecision.*

| | Within-run ($N = 30$) | | | Between-run ($N = 20$) | | |
|---|---|---|---|---|---|---|
| Analyte | $\bar{x}$ | SD | CV (%) | $\bar{x}$ | SD | CV (%) |
| Glucose (mmol/l) | 13·3 | 0·15 | 1·2 | 13·4 | 0·34 | 2·6 |
| | 6·6 | 0·06 | 1·0 | 6·7 | 0·22 | 3·3 |
| | 3·1 | 0·07 | 2·2 | 3·2 | 0·07 | 2·4 |
| Total protein (g/l) | 71·1 | 0·30 | 0·4 | 68·3 | 2·10 | 3·1 |
| | 48·1 | 0·77 | 1·6 | 60·1 | 1·90 | 3·2 |
| | 36·9 | 0·48 | 1·3 | 45·6 | 1·60 | 3·5 |
| Creatinine (mmol/l) | 278 | 1·76 | 0·6 | 266 | 14·14 | 5·3 |
| | 153 | 1·06 | 0·7 | 147 | 8·84 | 6·3 |
| | 79 | 0·50 | 0·6 | 77 | 4·42 | 5·9 |
| AST (U/l) | 78·5 | 0·64 | 0·8 | 77·4 | 0·99 | 1·2 |
| | 41·5 | 0·50 | 1·2 | 41·6 | 0·75 | 1·8 |
| | 20·6 | 0·49 | 2·3 | 20·8 | 0·93 | 4·4 |
| Alkaline phosphatase (U/l) | 321 | 3·08 | 0·9 | 283 | 11·79 | 4·1 |
| | 190 | 1·34 | 0·7 | 168 | 7·66 | 4·5 |
| | 103 | 1·38 | 1·3 | 91 | 4·65 | 5·1 |

*Table 5. Carry-over.*

| Analyte | Level | $N_1$ | $\bar{x}_1$ | $SD_1$ | $N_2$ | $\bar{x}_2$ | $SD_2$ | F calculated |
|---|---|---|---|---|---|---|---|---|
| Glucose (mmol/l) | High (H) | 120 | 13·4 | 0·43 | 69 | 13·4 | 0·46 | 1·14 |
| | Medium (M) | 60 | 6·7 | 0·23 | 40 | 6·7 | 0·23 | 1·00 |
| | Low (L) | 120 | 3·1 | 0·15 | 69 | 3·1 | 0·14 | 1·16 |
| Total protein (g/l) | H | 120 | 68·2 | 2·20 | 69 | 68·2 | 2·30 | 1·09 |
| | M | 60 | 60·3 | 1·90 | 40 | 60·4 | 1·87 | 1·03 |
| | L | 120 | 45·9 | 2·10 | 69 | 45·8 | 1·90 | 1·22 |
| Creatinine (mmol/l) | H | 120 | 267·8 | 14·14 | 69 | 267·8 | 14·14 | 1·00 |
| | M | 60 | 144·9 | 9·72 | 40 | 144·9 | 10·16 | 1·09 |
| | L | 120 | 76·9 | 4·42 | 69 | 76·9 | 4·95 | 1·25 |
| AST (U/l) | H | 120 | 77·7 | 1·09 | 69 | 77·9 | 1·21 | 1·23 |
| | M | 60 | 41·6 | 0·84 | 40 | 41·6 | 0·84 | 1·00 |
| | L | 120 | 20·7 | 0·69 | 69 | 20·7 | 0·60 | 1·32 |
| Alkaline phosphatase (U/l) | H | 120 | 284·1 | 11·70 | 69 | 284·8 | 11·80 | 1·01 |
| | M | 60 | 168·6 | 7·44 | 40 | 168·5 | 7·51 | 1·01 |
| | L | 120 | 91·3 | 4·38 | 69 | 90·9 | 4·69 | 1·45 |

$SD_1$ = Standard deviation for all the values ($N_1$) including the effects of contamination.
$SD_2$ = Standard deviation for values ($N_2$) without the effects of contamination.

*Table 6. Regression analysis of results obtained by EPOS 5060 and Ultrolab-Aurora.*

| Analyte | No. of pairs | Slope | SD/Slope | Intercept | SD of intercept | *r* |
|---|---|---|---|---|---|---|
| Glucose | 100 | 1·14 | 0·035 | − 8·05 | 4·80 | 0·956 |
| Total protein | 103 | 0·89 | 0·052 | +10·61 | 3·60 | 0·863 |
| Creatinine | 103 | 1·06 | 0·040 | − 0·09 | 0·03 | 0·935 |
| AST | 106 | 0·45 | 0·014 | +32·13 | 0·75 | 0·955 |
| Alakaline phosphatase | 109 | 0·61 | 0·010 | −27·59 | 2·08 | 0·987 |

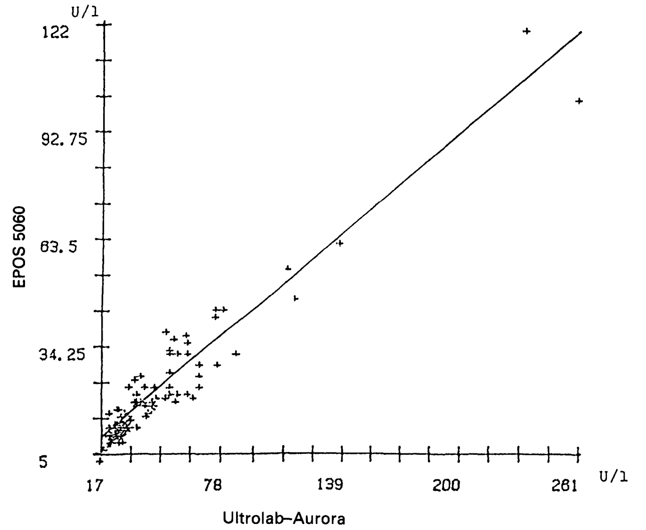

*Figure 3. Comparison of results obtained by two instruments: Regression line for AST (IFCC method).*

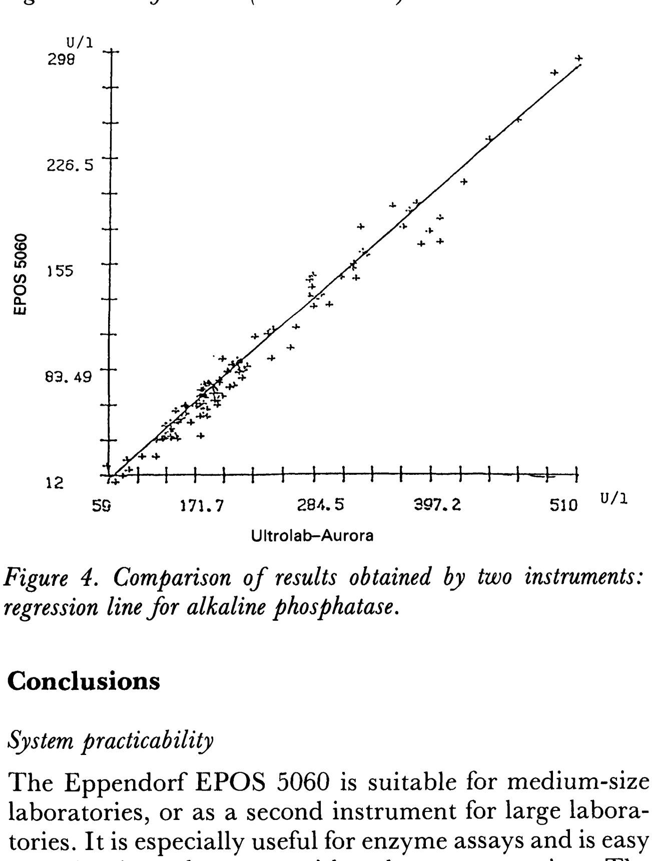

*Figure 4. Comparison of results obtained by two instruments: regression line for alkaline phosphatase.*

### Conclusions

*System practicability*

The Eppendorf EPOS 5060 is suitable for medium-size laboratories, or as a second instrument for large laboratories. It is especially useful for enzyme assays and is easy to maintain and operate with a short start-up time. The only external connection needed is a power supply. A waste container needs to be emptied periodically when indicated by an alarm. No special training is required for users. Any commercial reagents may be used and method change is not possible to introduce stat samples.

It is provided with an outline for the on-line linking to a central ordinator. Alarm flags appear with the results, giving information on linearity, and level of substrate for the central computer is provided.

*Analytical modules evaluation*

Photometric imprecision increases with decreasing absorbances, as expected, with coefficients of variation up to 1·0% for absorbances lower than 0·2.

The photometric linearity is acceptable, with some variation at low absorbances. The drift was negligible during the first 30 min, increasing slightly up to 12 h.

The pipette delivery system has acceptable imprecision, except when operating with less than 2 μl.

*Working conditions system evaluation*

There is no carry-over between samples. The imprecision for enzyme measurements is low and it is acceptable for the other analytes. Relative inaccuracy for total protein is not good, perhaps due to a calibration problem, since the reference instrument was calibrated with lyophilized serum, while an aqueous standard was used for the EPOS 5060. This aspect needs further study. The precision of the sample dilution system was found to be unsatisfactory but this problem has been resolved with new software. It will be the subject of a further evaluation.

### References

1. Galimany, R., Alsina, M. J., Biosca, C., Calvet, M., Castiñeiras, M. J., Esquerdo, E., Fuster, M., Lema, F., Martinez, M., Mateo, J., Navarro, J. M. and Paz, M., Protocolo de evaluación de analizadores automáticos. Evaluación de los módulos analíticos. *Boletın Informativo S.E.Q.C.*, **34** (1986), 3–17.
2. Galimany, R., Alonso, J. R., Barrio, J. M., Castiñeiras, M. J., Esquerdo, E., Fernández, E., García-Merlo, S., Lema, F., Mateo, J., Paz, M. and Roura, F., Protocolo de evaluación de analizadores autométicos. Evaluación preliminar. *Boletín Informativo de la S.E.Q.C.* (1980).
3. Grafmeyer, D. and Dingeon, B., Protocole etude de la partie photométrique des analyseurs biochimiques. *I.S.B.*, **6** (1984), 376–378.
4. Vanderlinde, R. R., Richards, A. H. and Kowalski, P., Linearity and accuracy of ultraviolet and visible wavelength photometers: an interlaboratory survey. *Clinica Chimica Acta*, **61** (1975), 39–46.
5. Geary, T. D., Procedures for checking the performance of samplers dispensers and diluters. *Clinical Biochemistry reviews*, **2** (1981), 64–66.

